# Autonomic neuropathy in dialysis patients – investigations with a new symptom score (COMPASS 31)

**DOI:** 10.1186/s12882-024-03691-y

**Published:** 2024-08-08

**Authors:** Catharina Verena Schramm, Michael Christoph Schramm, Markus Trautner, Michael Hinz, Steffen Mitzner

**Affiliations:** 1 Zentrum für Innere Medizin, Medizinische Klinik 1, Julius-Maximilians-Universität Würzburg, Oberdürrbacher Str. 6, Würzburg, 97080 Germany; 2 Medizinische Klinik und Poliklinik IV, LMU Klinikum, LMU München, Ziemssenstraße 5, München, 80336 Germany; 3 Klinik und Poliklinik für Innere Medizin, Abteilung für Nephrologie, Universitätsklinikum Rostock, Ernst-Heydemann-Str. 6, Rostock, 18057 Germany

**Keywords:** Autonomic neuropathy, COMPASS 31 (composite autonomic symptom score), Diabetes mellitus, Dialysis patients

## Abstract

**Background:**

Symptoms of autonomic neuropathy (AN) are common in patients with diabetes and advanced renal disease. As yet different domains of autonomic neuropathy cannot be detected by a singular laboratory or invasive test. COMPASS 31, a new self-assessment test, has shown reliable results not only in cardiac autonomic neuropathy but also in different sub-domains when judging manifestation of AN by scores.

**Methods:**

One hundred eighty-three patients with or without diabetes were enrolled, one hundred nineteen of them were treated with permanent dialysis therapy (HD), sixty-four patients served as controls (eGFR > 60 ml/min.) Using COMPASS 31 different symptoms of AN were assessed (orthostatic intolerance, vasomotor, secretomotor, gastrointestinal, bladder, pupillomotor changes) and transferred into AN-scores.

**Results:**

AN was more pronounced in dialysis patients compared with controls (AN-score 27,5 vs. 10,0; *p* < 0,01). These differences were present also in every sub-domain of AN (orthostatic intolerance, vasomotor, secretomotor, gastrointestinal, bladder, pupillomotor changes; *p* < 0,05 for all sub-domains). In diabetic patients there was a strong correlation between symptoms of AN and diabetes duration (correlation coefficient *r* = 0,45, *p* < 0,001). Current glycemic control (HbA1c), body mass index (BMI), sex, and height had no influence on AN when comparing dialysis patients and controls. C-reactive protein (CRP) showed a positive linear correlation with AN-scores (correlation coefficient *r* = 0,21; *p* < 0,05).

**Conclusion:**

Symptoms of AN are more pronounced in dialysis patients not only in total but also in all different domains of neuropathic changes. Longlasting diabetic disease promotes development of AN, as duration of diabetes was positively correlated with AN. Future longitudinal studies might help to identify the high cardiovascular and mortality risk in dialysis patients by the easy-to-use COMPASS 31 without need of invasive and time-spending methods for diagnosing AN.

## Introduction

AN is a common complication in long-term diabetes and chronic kidney disease. It is characterized by imbalance of sympathetic and parasympathetic signals resulting in disturbances of different organ systems with reduction of quality of life [1, 2]. Cardiovascular autonomic neuropathy (CAN) indicates adverse cardiovascular events and is a strong risk factor for increased mortality rate [3, 4]. Also other subtypes of autonomic disorders contribute to AN (orthostatic intolerance, vasomotor, pupillomotor, secretomotor, or gastrointestinal changes etc.). Alterations of these subtypes of autonomic disturbances and their impact on mortality, however, have not been investigated as yet in a comprehensive manner in dialysis patients.

Typical clinical tests of AN include tilt-table testing (orthostatic dysregulation), Valsalva maneuver, sudomotor testing and others [5]. Further invasive diagnostic methods are available, including nerve biopsies, electrophysiological techniques, and others. These tools are typically hospital- or research-based, more invasive, time-spending and not well accepted by patients.

**Fig. 1:**
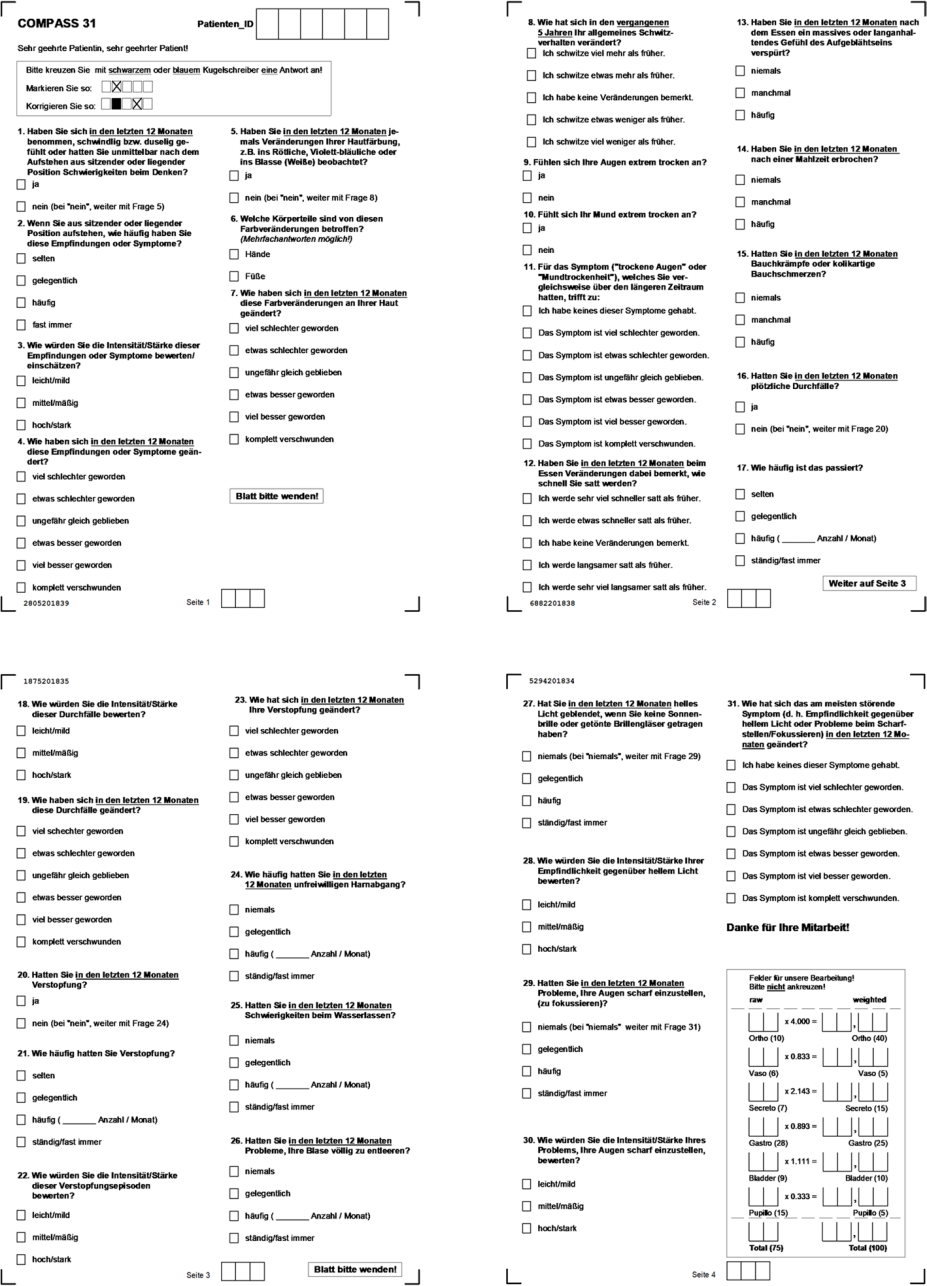
Questions and possible answers of the COMPASS 31 self assessment test regarding various domains of neuropathic changes; last page: calculation of weighted values from raw values by application of different multipliers

In the last years COMPASS 31 (shown in Fig. 1) has been developed to assess AN [6]. The self assessment method is a concise and statistically robust instrument based on the well established autonomic symptom profile ASP. It is considered suitable for widespread use in autonomic neuropathy research and practice [7, 8].

We tried to identify if—beyond testing singular AN domains only—different sub-domains of AN (orthostatic intolerance, vasomotor changes, pupillomotor, secretomotor, bladder, or gastrointestinal changes) could be simultaneously detected in dialysis patients by this new test and if there are clinical correlations with diabetes duration, chronic inflammation, BMI and other relevant clinical parameters.

## Methods

After written declaration of consent 183 patients with and without diabetes mellitus were enrolled in our study, that was approved by the ethical commitee of the university of Rostock (Nr. A2015-0176). In the dialysis group only patients with permanent dialysis therapy (hemodialysis only) were included; patients with eGFR > 60 ml/min./1,73 m^2^ served as controls. eGFR was estimated using the Chronic Kidney Disease Epidemiology Collaboration (CKD-EPI) creatinine equation. Acute renal failure, specific neurological diseases, intoxication, alcohol abuse, age under 18 or cognitive impairment resulted in exclusion from the investigation.

COMPASS 31 is a self-assessment test that is well defined and shows good reliability and retest values as well. It is broadly applicable, easy to administer in a short amount of time, and based on a scientific approach. Results from 31 questions were entered and transformed to raw values (depending on extent and severity of symptoms) and finally transferred to weighted values. The obtained values resulted in differing degrees of neuropathic symptoms (AN-scores) ranging from 0 to 100 (0 no symptoms, 100 maximum expression). Interpretation and performance of COMPASS 31 are described in detail elsewhere [6, 9]. Blood samples were taken immediately before dialysis treatment. At the same day self assessment tests were performed and other clinical and epidemiological parameters (age, gender, height, BMI, etc.) were recorded. Data concerning presence of diabetes, duration of dialysis therapy, and duration of diabetes disease were collected from the patients’ medical records and by direct patient questioning.

Statistical analysis was performed using SPSS („Statistical package for the social sciences “). In normally distributed parameters t-Test was used to detect group differences, in non normally distributed values we used U-Test of Mann and Whitney. Strength of correlation was determined by Pearson correlation coefficient. Significance was assumed with a value of *p* < 0.05.

## Results

Table 1 shows epidemiological and clinical data of dialysis patients and controls with no differences for sex, height, and age. Dialysis patients’ weight was significantly lower compared with controls (76,6 vs. 84,5 kg), and diabetes duration was longer due to the mostly longer time period of diabetes disease preceding the period of chronic dialysis therapy (23,4 vs. 12,5 years). Changes in laboratory values showed typical differences between dialysis group and controls with higher values for potassium, urea and creatinine and slightly lower levels for sodium, eGFR and hemoglobin, the latter indicating renal anemia in dialysis patients. There were no differences for current HbA1c, while C-reactive protein was markedly elevated in dialysis patients (9,3 vs. 3,2 mg/dl). A remarkable group difference was found for AN-scores, the value indicating autonomic polyneuropathic changes of different organ systems. Much higher values were observed in dialysis patients in comparison with controls (27,5 ± 15,6 vs 10 ± 10,3; *p* < 0.01). Additionally, AN-scores were obtained from scores of different subtypes of autonomic neuropathic changes (orthostatic intolerance, vasomotor, secretomotor, gastrointestinal, bladder, pupillomotor). When assessing AN separately for each subtype, the differences between dialysis patients and controls were significant in each single subtype, too (Table 1).

**Table 1:**
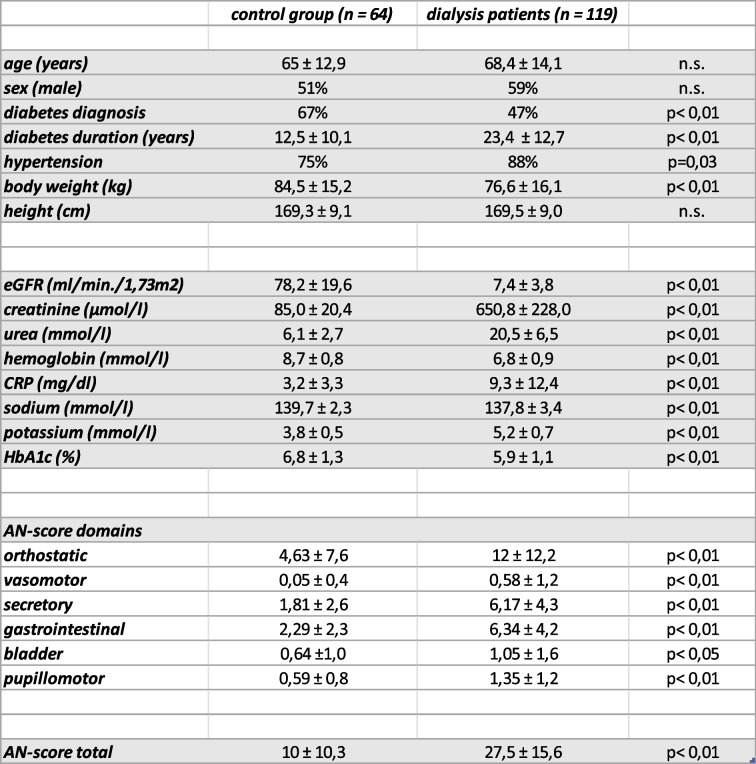
Epidemiological data, laboratory values, AN-score of different domains and total AN-score in control group and dialysis patient group; significant differences are indicated by *p*-values, *n.s.* means not significant

**Fig. 2:**
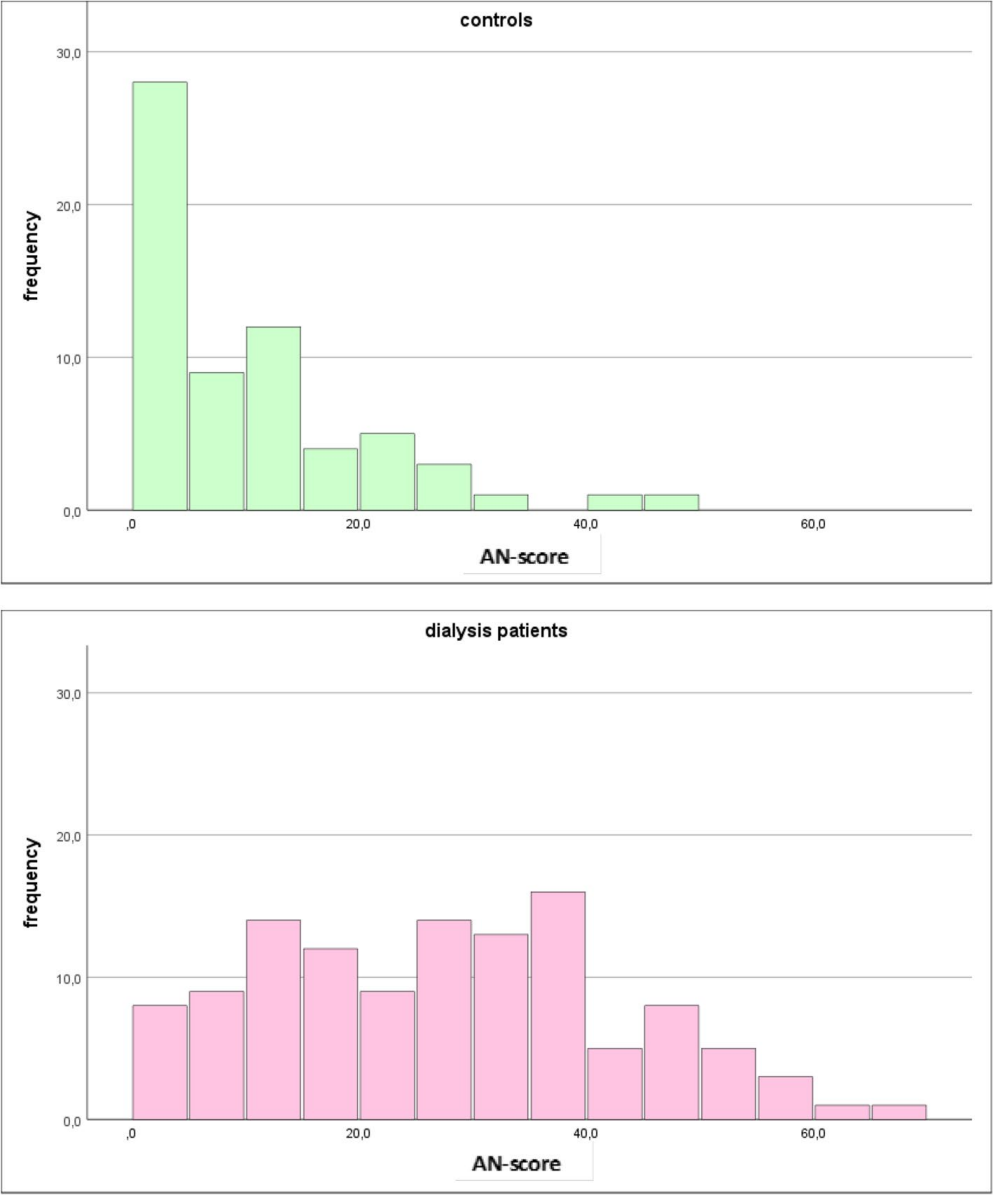
Level and frequency distribution of AN-scores for the control group (green, *n* = 64; 10,0 ± 10,3) and the group of dialysis patients (red, *n* = 119; 27,5 ± 15,5) with mean and standard deviation

Level and frequency distribution of AN-scores for the control group (green) and the group of dialysis patients (red) are shown in Fig. 2. In dialysis patients symptoms of AN were more pronounced with markedly higher levels of AN-scores.

**Fig. 3:**
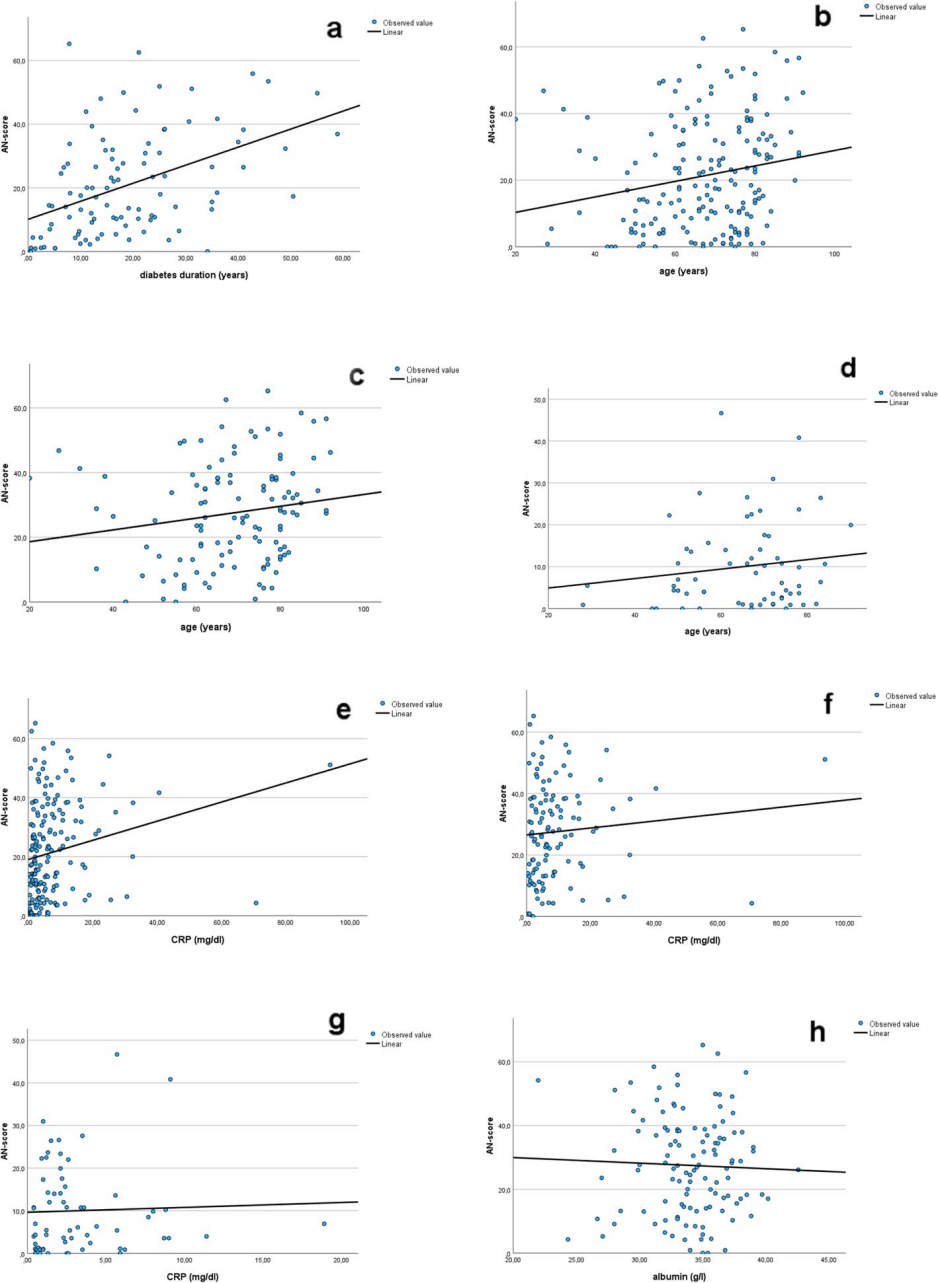
**a** AN-scores of all patients plotted against duration of diabetes showing linear and positive correlation (correlation coefficient *r* = 0,45, R^2^ = 0,20, standard error of the estimator = 14,42, *p* < 0,001. **b** AN-scores of all patients plotted against age showing linear and positive correlation (correlation coefficient *r* = 0,19, R^2^ = 0,038, standard error of the estimator = 15,9, *p* < 0,05). **c** AN-scores of dialysis patients plotted against age showing linear but not significant correlation (correlation coefficient *r* = 0,165, R^2^ = 0,027, standard error of the estimator = 15,4, *p* = 0,07). **d** AN-scores of controls plotted against age showing linear but not significant correlation (correlation coefficient *r* = 0,141, R^2^ = 0,02, standard error of the estimator = 10,3, *p* = 0,26. **e** AN-scores of all patients plotted against CRP showing linear and positive correlation (correlation coefficient *r* = 0,21, R^2^ = 0,044, standard error of the estimator = 15,9, *p* < 0,05. **f** AN-scores of dialysis patients plotted against CRP (mg/dl) showing no significant correlation (correlation coefficient *r* = 0,09, R^2^ = 0,008, standard error of the estimator = 15,6, *p* > 0,05. **g** AN-scores of controls plotted against CRP (mg/dl) showing no significant correlation (correlation coefficient *r* = 0,037, R^2^ = 0,001, standard error of the estimator = 10,4, *p* > 0,05. **h** AN-scores of dialysis patients plotted against albumin concentration in the serum showing no linear correlation (correlation coefficient *r* = 0,032, R^2^ = 0,001, standard error of the estimator = 15,6, *p* > 0,05)

Duration of diabetic disease is associated with development of long-term complications. AN-scores of all patients are plotted against duration of diabetes in Fig. 3a, showing linear and positive correlation (correlation coefficient *r* = 0,451, R-Quadrat = 0,204, standard error = 14,42, *p* < 0,001). Higher AN-scores are seen more frequently in patients with longer duration of diabetes and consecutively more pronounced late complications of the disease.

Glycemic control was determined by HbA1c and no correlation was found between the present glycemic control and the concurrent AN-score when comparing dialysis patients and controls. While we found no correlation between AN-scores and albumin concentration in dialysis patients, sex, weight, height and BMI (Fig. 3h; Table 2), there was a significant correlation between AN-scores and patients’ age (Fig. 3b). After analyzing this difference separately for controls and dialysis patients statistical significance was lost due to reduced sample size (Fig. 3c and d). CRP as a marker of chronic inflammation was elevated in dialysis patients (Table 1; 9,3 vs. 3,2 mg/dl), and also positively correlated with AN-scores (Fig. 3e). This correlation was seen also when regarding CRP and AN-score in controls and dialysis patients separately. Statistical significance, however, was narrowly missed presumably due to reduced numbers in the groups (Fig. 3f and g).

**Table 2:** Correlation of total AN-score with sex, weight, height and BMI; significant differences are indicated by *p*-values, *p* < 0,05 means not significant

**AN-score**	**Sex**	**Weight (kg)**	**Height (cm)**	**BMI**
correlation (Pearson)	,012	-,128	-,050	-,120
significance (2-sided)	,867	,084	,502	,107
N	183	183	183	183

## Discussion

Autonomic neuropathic changes are frequent in patients with diabetes and also in those suffering from chronic kidney disease [3, 10, 11]. Especially cardiac autonomic neuropathy (CAN) may result in severe consequences such as sudden death, myocardial ischemia and overall increased mortality [2, 4]. Using COMPASS 31, a new self-assessment test, that is well defined and shows good reliability, we could document much higher AN-scores in dialysis patients compared with controls (Fig. 2). As there is a strong correlation between AN-score and diabetes duration (Fig. 3a) and as in the dialysis patients’ group diabetes duration is longer than in controls (23,4 vs. 12,5) this potential effect should be taken into account as a possible limitation. However, even after dichotomization of diabetes duration for dialysis patients’ group (AN-score lower or higher as the median of 16 years), there was still a significant difference for each of the groups compared with controls (group with diabetes duration < 16 years: *p* < 0,05; mean for AN-score 25,8 vs. 8,4; group with diabetes duration > 16 years; *p* < 0,01; mean for AN-score 29,7 vs. 15,3). Increased AN-scores were found not only for cardiac autonomic alterations, which mostly have been in the focus of preceding investigations, but also for neuropathic disorders of other affected organ systems (secretomotor, gastrointestinal, bladder, pupillomotor subtypes). To our knowledge this the first investigation in dialysis patients involving comprehensively these subtypes of neuropathic changes besides signs of CAN by a self assessment test.

Incidence and progression of AN in dialysis patients at least partially are due to uremic toxins [12, 13]. Besides uremic toxins also advanced glycation endproducts (AGEs) play an important role in the genesis of autonomic neuropathy, especially in patients with diabetes disease [14]. Moreover various reactive oxygen metabolites and oxidative stress contribute to the development of AN, as well as disturbances in the polyol and other pathways [3]. Abovementioned substances partially unfold their neurotoxic effects as a consequence of their insufficient removal by dialysis therapy. If dialysis therapy is able to improve AN is not generally proven. Some authors report partial regression of AN after initiation of dialysis therapy while others could not confirm a clear correlation between time period after starting dialysis therapy and extent of autonomic neuropathic symptoms [15, 16]. Patients with pronounced autonomic neuropathic disorders are at high risk for cardiovascular events and death. High AN-scores eventually could enable us to treat these patients more intensively. With respect to renal replacement therapy this could possibly result in hemodiafiltration treatment instead of hemodialysis treatment.

In the multimorbid collective of dialysis patients many of them are suffering not only from abovementioned metabolically induced changes but also from ongoing vascular damage with long-lasting diabetes. This may further aggravate AN and additionally explain the highly significant correlation between AN-score and overall duration of diabetes disease in our study cohort.

Current HbA1c was not associated with AN-scores in our collective. It can be hypothesized that not all dialysis patients had good monitoring of diabetes disease all the time before starting renal replacement therapy. On the other hand, it can be assumed in some cases that glycemic control was even improved with the initiation of dialysis therapy, at a moment where longlasting negative effects on autonomic nerve function had resulted in severe AN. Low HbA1c in dialysis patients partially might be attributed to compromised appetite, reduced body weight and thus improved insulin resistance (lower body weight in this group). Furthermore dialysis patients are usually seen three times a week and thus have a frequent and tight relation with their nephrologist. The effect of improved medical care in improving glycemic control in these patients should not be underestimated.

The missing correlation of AN-score with BMI may be explained by the fact that most dialysis patients are suffering from long existing neuropathic changes, while on the other hand during dialysis therapy loss of appetite, malnutrition and reduced general condition are increasing. Thus, potential influences of BMI on AN-score may change with time and possible correlation may disappear.

In some studies height was correlated with severity of AN and attributed to increasing length of nerve fibers with greater body height. Other authors, however, could not find such an association in a population study, and are in accordance with our results [17, 18]. Age and AN-score are positively correlated, as shown in Fig. 3b. Statistical significance, however, was narrowly lost when analyzing both control group and dialysis patient group separately (Fig. 3c and d).

It is well known that chronic inflammation is enhanced in chronic kidney disease and especially in dialysis patients [19–22]. This could be confirmed in our study and we additionally found a positive correlation with AN-score. To what extent this association of CRP and AN-score is due to inflammation, vascular changes or mediators of other pathways can not be answered by our study.

A limitation of our study might be, that in dialysis patients the anuric state may impact the subdomain “bladder”. As anuria was present in only 14% of dialysis patients and due to the fact that there was still diuresis of more than 500 ml in 55% and more than 1000 ml in 31% of the dialysis patients, we think this is in total acceptable. Also the AN-score for bladder is weighted only with a factor of 0.33. Moreover the self assessment test was done before starting of the daily dialysis treatment. The questionaire includes time-dependency of dry eye problems and of sweating in general (have you recognized an increased frequency of dry eyes or mouth in the past?). This would detect developing of polyneuropathic symptoms in time course. Nevertheless there could be effects of actual hydration and dry weight changes, however, more likely in orthostatic and vasomotor problems. This should be taken into account but remains a difficulty in most studies in dialysis patients. Generally also antihypertensive treatment may influence changes in these domains.

Future cross sectional and longitudinal studies with higher case numbers might help to use results from the COMPASS 31 tool for better detection of autonomic neuropathic changes in the variety of affected organ systems.

## Conclusion

AN is frequent and more pronounced in dialysis patients than in patients with normal or slightly reduced renal function. This could be shown for the first time for different items of neuropathic alterations (orthostatic intolerance, vasomotor, secretomotor, gastrointestinal, bladder, pupillomotor) by a self assessment test suitable for widespread use. Duration of diabetes was positively correlated with symptoms of AN, while current glycemic control, gender and BMI had no impact. There is a positive linear correlation between AN and CRP, also known as an indicator of increased mortality. Future longitudinal studies might help to identify the high cardiovascular and mortality risk in dialysis patients by the easy-to-use COMPASS 31 test without need of invasive and time-spending methods.

## Data Availability

The datasets used and/or analysed during the current study are available from the corresponding author on reasonable request.
